# A review of novel analytical diagnostics for liquid biopsies: spectroscopic and spectrometric serum profiling of primary and secondary brain tumors

**DOI:** 10.1002/brb3.502

**Published:** 2016-06-29

**Authors:** Katie Spalding, Ruth Board, Timothy Dawson, Michael D. Jenkinson, Matthew J. Baker

**Affiliations:** ^1^ WestCHEMDepartment of Pure and Applied ChemistryTechnology & Innovation CentreUniversity of Strathclyde99 George StreetGlasgowG1 1RDUK; ^2^ Rosemere Cancer CentreLancashire Teaching Hospitals NHS TrustRoyal Preston HospitalSharoe Green LanePrestonPR2 9HTUK; ^3^ NeuropathologyLancashire Teaching Hospitals NHS TrustRoyal Preston HospitalSharoe Green Lane NorthPrestonLancashirePR2 9HTUK; ^4^ The Walton Centre for Neurology and NeurosurgeryThe Walton Centre NHS Foundation TrustLower LaneFazakerleyLiverpoolL9 7LJUK

**Keywords:** Cancer, infrared, serum, spectrometry, spectroscopy

## Abstract

**Introduction:**

Spectroscopic and spectrometric analysis of biological samples is regarded as quick, cost effective, easy to operate, and spectroscopic sample preparation involves minimal sample preparation.

**Results:**

Techniques like infrared (IR) spectroscopy, surface‐enhanced laser desorption/ionization (SELDI)‐mass spectroscopy (MS), and matrix‐assisted laser desorption/ionization (MALDI) ‐MS could enable early diagnosis of cancer, disease monitoring, and assessment of treatment responses allowing refinement, if required.

**Discussion:**

Carrying out analytical testing within outpatient clinics would dramatically cut the time spent by patients attending different appointments, at different locations, save hospital time and resources but importantly would theoretically enable a reduction in mortality and morbidity. While the advantages of such a prospect seem obvious, this review aims to evaluate the use of human serum spectroscopic and spectrometric analysis as a diagnostic tool for brain cancers, creating a platform for the future of cancer diagnostics.

## Introduction

With nearly 9400 new cases of brain tumors diagnosed each year in the UK and over half of them resulting in death, the demand for a rapid, noninvasive diagnostic test permitting early detection is as vital and evident as ever (Cancer Research UK).

**Figure 1:**
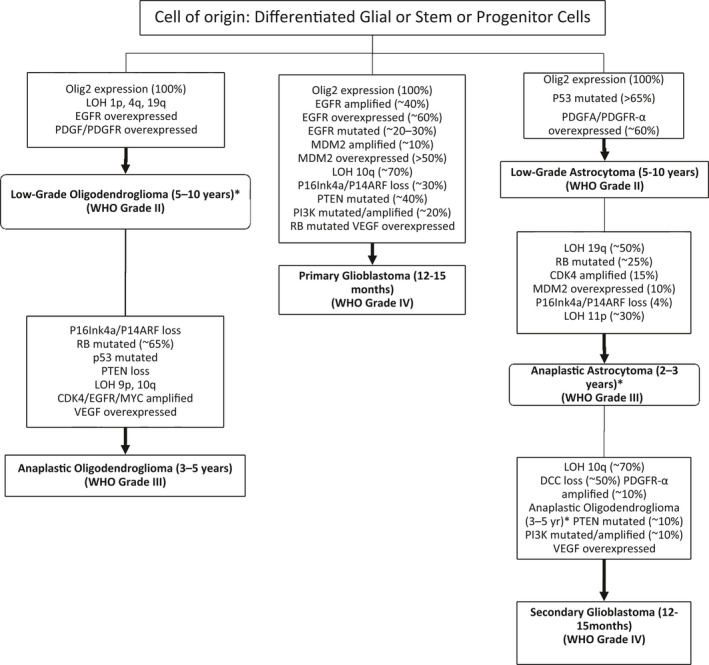
Pathways leading to the diagnosis of malignant gliomas. [Figure adapted from (Wen and Kesari 2008)].

Brain tumors form when normal cells within the brain mutate, grow uncontrollably and form a mass. Brain tumors are stratified into increasing grades of malignancy determined by how they are likely to grow, the likelihood of reoccurrence, and the likely best treatment, highlighted in Figure 1 (Wen and Kesari 2008). Gliomas occurring from the glial cells within the brain and central nervous system (CNS) (Hands et al. 2014) are the most common primary brain tumor classified by World Health Organisation (WHO) (Louis et al. 2007). Gliomas are often diagnosed late, are hard to treat, and the prognosis is poor. Generally, only 40% of patients with brain tumors are alive more than 1 year after diagnosis (Cancer Research UK). Traditional treatment involves surgery, radiation therapy, and chemotherapy but even with an extensive treatment plan survival is poor as symptoms are often diagnosed too late. The most common tumor types; astrocytomas, ependymomas, and oligodendrogliomas have very different outcomes.

Grade I and II astrocytomas are slow growing; often grade I tumors can be completely removed through surgery, producing a good outlook. Grade II tumors have moderate survival rates, 40% of those diagnosed survive for more than 10 years; nevertheless, they can develop into higher grade, harder to treat tumors the longer they go undiagnosed and untreated. Glioblastoma (GB), a grade IV rapidly growing astrocytoma, often leads to patients living for less than 1 year, only 6% survive for more than 5 years (Hands et al. 2014). Identification and analysis, within clinics, of characteristic biomarkers/biosignatures present within an individual's blood would enable the early detection of grade II, III, and IV gliomas, allowing for earlier treatment, leading to improved patient mortality, morbidity, and quality of life (Cancer Research UK; The Brain Tumour Charity).

Despite extensive therapeutic development resulting in a variety of treatments, brain tumors still result in low survival rates. A target area for improvement is early diagnosis. Patients often attend their GP practice on multiple occasions (in 2013, 38% of brain tumor patients reported to their GP more than five times prior to diagnosis) (The Brain Tumour Charity, 2014), and are then referred for magnetic resonance imaging (MRI) and/or computed tomography (CT). These results are then confirmed by histological assessment of a surgical specimen following a brain biopsy; however, diagnosis is often made too late for curative therapy (Bashour et al. 2015; Shanthakumar and Ganeshkumar 2015). A methodology capable of early diagnosis or being able to monitor high‐risk patients would prove to be an extremely valuable tool.

Spectroscopic and spectrometric analysis of biological samples is regarded as quick, cost effective, easy to operate and spectroscopic sample preparation involves minimal sample preparation. Techniques like infrared (IR) spectroscopy, surface‐enhanced laser desorption/ionization (SELDI)‐mass spectroscopy (MS), and matrix‐assisted laser desorption/ionization (MALDI) ‐MS could enable early diagnosis of cancer, disease monitoring, and assessment of treatment responses allowing refinement, if required. Carrying out analytical testing within outpatient clinics would dramatically cut the time spent by patients attending different appointments, at different locations, save hospital time and resources but importantly would theoretically enable a reduction in mortality and morbidity (Cancer Research UK). While the advantages of such a prospect seem obvious, this review aims to evaluate the use of human serum spectroscopic and spectrometric analysis as a diagnostic tool for brain cancers, creating a platform for the future of cancer diagnostics.

Analytical research has focused on identifying a set of biomarkers indicative of brain cancer that can be identified during a relatively noninvasive test (Jung et al. 2007; Kalinina et al. 2011; Marella 2013; Oslobanu and Florian 2015). Blood is a favored sample medium due to the ease of obtaining samples from patients and highly discriminatory components contained therein. Serum is the liquid remaining when whole blood is left to clot and then processed via a centrifuge, which concentrates the protein and peptide biomarkers. This short review examines infrared spectroscopy and mass spectrometry, underlining recent novel research to understand serum sample “biosignatures” to profile brain cancer.

## Mass Spectrometry

Mass spectrometry (MS) provides information on the molecular weight of a compound. Samples are ionized and then fragment in a characteristic manner, allowing the structure to be determined from a mass spectrum.

SELDI‐MS and MALDI‐MS are two spectrometry techniques that differ through the way the analyte is captured. They are the most common variations (Aebersold and Mann 2003) used for the analysis of biomolecules and have been used in proteomic profiling for the diagnosis, prognosis, and therapeutic monitoring of cancer.

The MALDI‐MS sample is mixed with an excess amount of matrix that readily absorbs ultraviolet (UV) light (Jung et al. 2007) and crystallizes under the influence of the vacuum. When the matrix is irradiated with short laser pulses, the ultraviolet light is absorbed causing the matrix to heat extremely quickly, causing vaporization of the matrix and the sample (Shimadzu). The analyte is ionized by charge transfer reactions with the matrix ions.

MALDI is regarded as being extremely useful for the identification of proteins and peptides. The analyzer usually coupled to MALDI is a TOF which measures the m/z ratio of molecules based on the time taken to reach a set distance (Kalinina et al. 2011). The ions created by the laser pulse accelerate toward the TOF due to the influence of an electric field. The potential difference is constant in relation to all the ions present, meaning either the ions with the higher charge or the smaller ions reach the detector first. Knowledge of this, as well as instrumental constants are used to calculate the m/z.

Despite the sample preparation used in SELDI being very unique, the technique is regarded as very similar to MALDI (Issaq 2002). The sample and the matrix are cocrystallized on the top of a target surface and a laser is used to ionize the sample. Reports have shown that protein profiles can be produced from serum samples as small as one microliter. SELDI‐TOF MS has enabled the discovery of potential diagnostic markers for cancers of the prostate (Cazares 1999), bladder (Vlahou et al. 2001), breast (Wulfkuhle 2001; Vlahou 2003), and ovaries (Petricoin 2002).

The protein chip array is the aspect of the instrumentation that separates SELDI from all other types of MS. They are made of chemical or biochemical surfaces that are designed to specifically retain the proteins of interest. Often, chemically active surfaces are used to retain whole groups of proteins, whereas biochemically active surfaces are used to interact with a single target protein. The analyzer used is a TOF mass spectrometer as explained above.

One of the main advantages of SELDI‐TOF MS is the fact that complex samples can be analyzed, making it particularly useful for the analysis of serum and other bio‐fluids.

There is a large interest in using SELDI‐TOF MS to develop a diagnostic tool for the early detection of disease. As mentioned above many clinical investigations have been completed to try and accomplish proteomic profiles of different types of cancer, including brain using SELDI‐TOF and bio‐fluid samples. This review will go on to discuss and consolidate such discoveries.

Multiple proof‐of‐principle studies have been completed. Zhang et al. (2007) used SELDI‐TOF MS to screen for serum biomarkers of astrocytoma, in which seven peaks were selected to build a decision tree, able to discriminate cancer from noncancer with sensitivities and specificities of 84.6% and 86.4%, respectively. Reporting a higher specificity than MRI, as a result of the inclusion of low molecular weight compounds not easily detected by traditional methods, led to Zhang discussing the usefulness of SELDI for suspect patients, where MRI findings were inconclusive (Kalinina et al. 2011). Azok et al. (2004) discovered four peaks, corresponding to low molecular weight compounds, in GBM serum samples that were elevated in comparison to normal patients. This supported the discussion of contrast‐enhanced tumor regions containing a higher number of low molecular weight peptides. Liu et al. (2005) screened a total of 22 biomarkers enabling the discrimination of glioma and benign tumors with sensitivities and specificities of 88.9% and 86.4%.

Analyzing serum samples using SELDI‐TOF MS, with the aim of screening for specific biomarkers could enable early detection as well as personalized treatment dependent on the tumor grade (Aebersold and Mann 2003; Popescu 2010). However, a single parameter enabling the detection of cancer is highly unlikely (Popescu et al. 2014) and it is thought that a biosignature would be most efficient due to the heterogeneity of the disease.

MALDI‐TOF MS allows the analysis of complicated sample mixtures with good sensitivities, reporting the detection of 400 polypeptides within a molecular mass range of 800–15,000 Da, in a 50 μL drop of serum, establishing it as a great technique for serum peptide profiling (Shimadzu). Novel technology was described by Villanueva et al. (2004) where MALD‐TOF MS was used to simultaneously analyze serum proteins; 1691 peptide masses were identified over the whole dataset. Refinement using a Wilcoxon–Mann *U*‐test enabled 274 peptide masses to be identified, differentiating GBM patients and controls, correctly classifying 96.4% of predicted samples as cancerous or not.

Further proof‐of‐principle research completed a decade later, 2013, Li et al. (2013) used magnetic beads to capture peptides within serum. Analysis with MALDI‐TOF MS, pattern recognition, and peak intensity comparisons of 11 peaks were used to classify different grades of glioma, but concluded that further validation was needed.

Evidently, several research reports have discussed the use of SELDI and MALDI to generate peptide profiles of glioma, with the work discussed being just a few. In reviewing the reports, peak combinations can be used to discriminate cancerous samples from noncancerous, but it can be seen from Table 1 that not all identify the same peaks. Further validation studies are required and the search for markers to act as screening tools is ongoing. The use of vibrational spectroscopy profiles could be more advantageous to identify cancer from serum samples, rather than specific, individual serum markers due to the heterogeneous disease profile of cancer.

**Table 1:** Identified peak combinations from various research projects

Researcher	No. identified	Peak combination (*m/z* ratios)
Zhang	7	2018, 4286, 6633, 7567, 8140, 8926, and 15115
Azok	4	2792, 4286, 4503, and 5813
Liu	22	9198.31, 22513.91, 22888.73, 2256.76, 23481.05, 23087.61, 4155.28, 2489.11, 2246.47, 2617.14, 15099.38, 22666.41, 29047.13, 14378.33, 24002.85, 2891.43, 14047.77, 2006.00, 14951.04,2267.22, 23672.58 and 22331.29
Liu	15	8214.77, 8926.76, 4815.11, 8612.23, 2082.19, 4299.87, 2103.54, 7764.82, 2368.19, 3226.97, 2389.55, 2021.78, 4469.09, 6457.054, 8702.416
Villanueva	274	Individual Peaks Not Stated
Li	11	1296.77, 2105.86, 2769.20, 2932.82, 4210.20, 4266.27, 4964.31, 5634.09, 6379.58, 6530.04, 8142.23

## IR Spectroscopy

Spectroscopy is the study of molecular structure through the emission, absorption, or scattering of light and each of these fundamental processes relate to a different form of spectroscopy. Spectroscopy can therefore be referred to as the study of the exchange of energy between electromagnetic radiation and matter. Electromagnetic radiation can be measured as packets of energy called photons which have specific energies and can be quantized (Smith and Dent 2005; Larkin 2009).

The sample is scanned using infrared radiation across a variety of frequencies, allowing many vibrational transitions to occur involving the absorption of light. Each molecular transition needs a different amount of energy to do so and create a characteristic signal, allowing the production of a unique spectrum which acts like a fingerprint of the analyte.

The use of IR spectroscopy in histopathology has been shown to be successful over the past decade, presenting the possibility of early diagnosis using less invasive surgical methods and real‐time monitoring (Bonnier et al. 2014). Spectroscopy is a measure of the interaction between light radiation and matter which is characteristic of the biomolecule being examined, allowing the production of a spectrum unique to the compound (Larkin 2009).

As with MS, the vast majority of currently published research involves proof‐of‐principle studies, highlighting the many advantages of using Fourier‐Transform IR (FTIR) spectroscopy to differentiate cancerous from noncancerous samples. FTIR spectroscopy coupled with data analysis has been reported to discriminate breast (Backhaus et al. 2010) and ovarian (Owens et al. 2014) cancers, as well as grade brain tumors from serum samples.

Gliomas are the most frequently encountered primary brain tumor in the UK and in 2014 Hands et al. (Azok et al. 2004) published work using ATR‐FTIR spectroscopy together with support vector machine (SVM) analysis to discriminate between brain tumors of differing types and grades. Results demonstrated that the analysis of a 1 μL drop of serum could produce high quality and reproducible spectra. The application of SVM analysis enabled analysts to distinguish between high‐grade, low–grade, and noncancerous serum samples. Principal component analysis (PCA) was also used to identify any variance between the three different serum grades, which were identified as a result of different protein, lipid, and DNA/RNA peaks. Whole serum was reported with the greatest specificity and sensitivity, tentatively assigned to cytokines and chemokines. Hands et al. had previously performed research demonstrating how cytokines and chemokines could be used as serum biomarkers to classify serum from glioma and serum from noncancer patients (Hands et al. 2013). This was the first time in which biochemical markers and their characteristic spectrochemical signals were used to diagnose gliomas from serum samples with sensitivities and specificities as high as 87.5% and 100%, respectively. Therefore, the diagnosis of gliomas could be achieved within 10 min of being placed on the ATR‐FTIR instrumentation.

**Figure 2:**
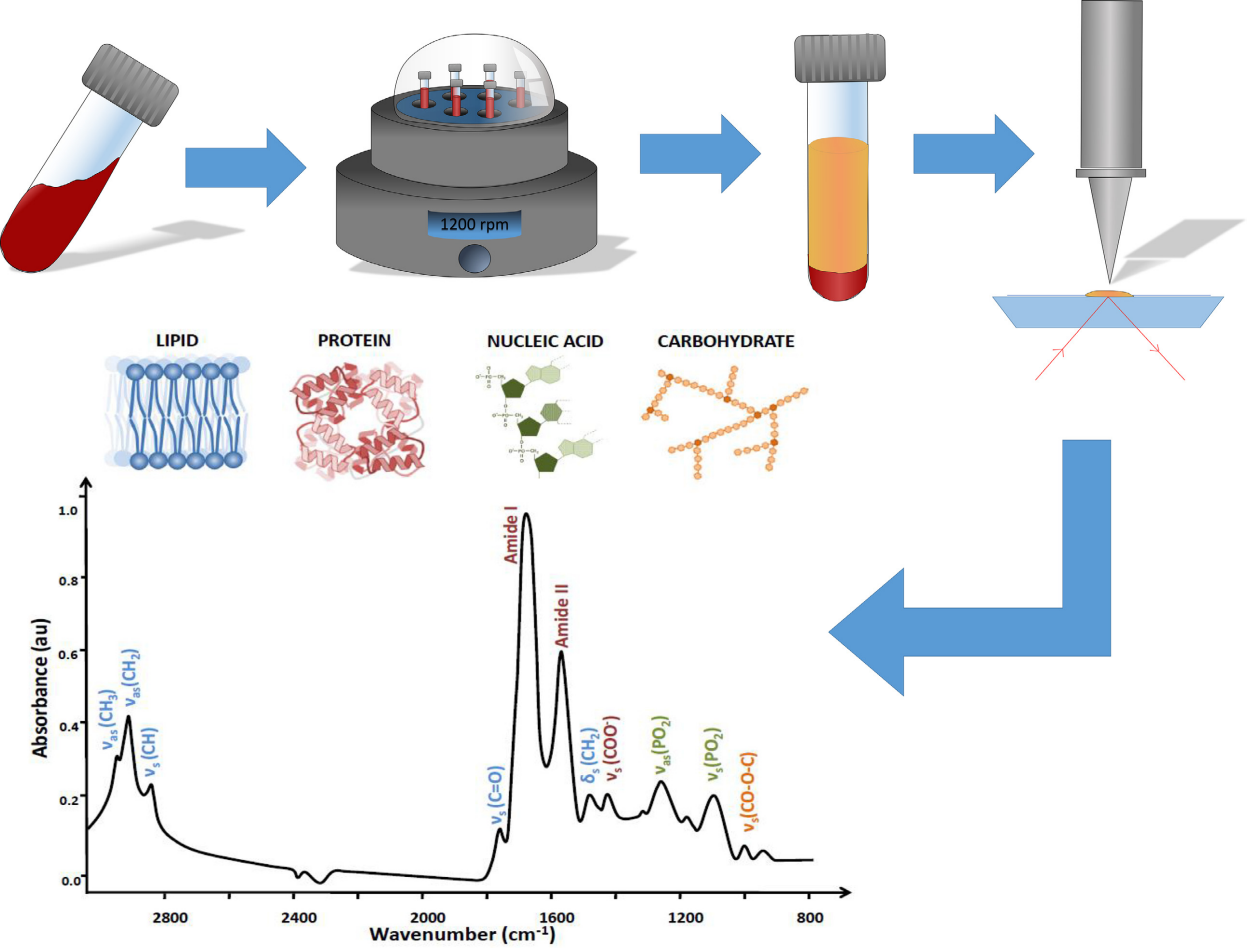
The process from the blood sample being obtained up until the acquired spectrum. Starting with the blood sample taken from the patient, which is then centrifuged to separate out the serum from all other components. The serum is then analyzed using ATR‐FTIR spectroscopy, which produces a spectrum allowing the protein, lipid, nucleic acid, and carbohydrate peaks to be seen. Data analysis follows which differentiates cancerous samples from healthy samples. Figure adapted from (Baker et al. 2014).

Infrared analysis is already used worldwide, creating a mature market for easy translation into routine clinics. Studies have reviewed the characteristics and advantages of the technique (Hughes et al. 2016); the proposed process is highlighted in Figure 2 (Baker et al. 2014).

The minimal sample preparation, the existence of hand held instrumentation, the simple operation, and the minimally invasive nature of the technique would undoubtedly enable analysis of serum samples in a clinical setting (Dorling and Baker 2013). Many of the research projects discussed have used relatively small sample populations and results have not progressed further than the laboratory—a large multi‐institutional study is urgently needed to move this research development into clinical practice.

## Clinical Translation

Currently the gold standard for the clinical validation of protein biomarkers are immunoassays such as ELISA, with MS and IR‐based approaches becoming an appealing prospect as a result of the capability at dealing with multiplexed sample with a high analytical specificity and sensitivity.

**Figure 3:**
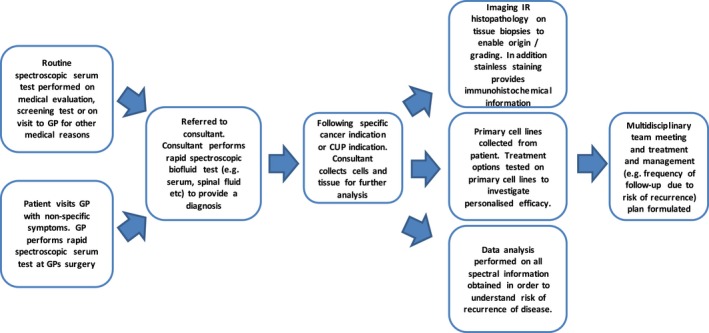
Schematic showing the potential use of serum spectroscopy within a clinical setting [Figure adapted from (Hughes and Baker 2016)].

However, the multiple advantages of using IR spectroscopy within clinics allow future research to involve validation and translation into clinical environments, taking into consideration all preanalytical factors that could jeopardize approval and limit clinical applications. Hand held instrumentation would allow spectra to be acquired within the clinic, significantly decreasing diagnosis time, improving patient treatment and management, and enable effective use of healthcare time and resources (Popescu 2010). Dorling and Baker, presented a schematic highlighting the potential for serum analysis using spectroscopy within a clinical environment, expanded further by Hughes et al. (2016) (Hughes and Baker 2016) – see Figure 3.

There are many sample requirements that must be taken into account in order to validate a research project. An appropriate target population of a significant size must be used, normally with sample sizes increasing, the closer the discovery gets to being ready for clinics. Robust statistical analysis of validation data of potential biomarkers reduces the chance of bias.

There are current challenges with translating serum spectroscopic diagnostics to the clinic, demonstrated by the majority of studies performed involving small‐scale laboratory‐based experiments. To enable clinical translation, studies based on large populations are necessary. Focusing on procedural and instrumental standardization would enable regulatory requirements to be met. However, when moving from the laboratory to large clinical trials, funding issues and gaining regulatory approval can create a valley of death which hinders translation of promising techniques.

**Figure 4:**
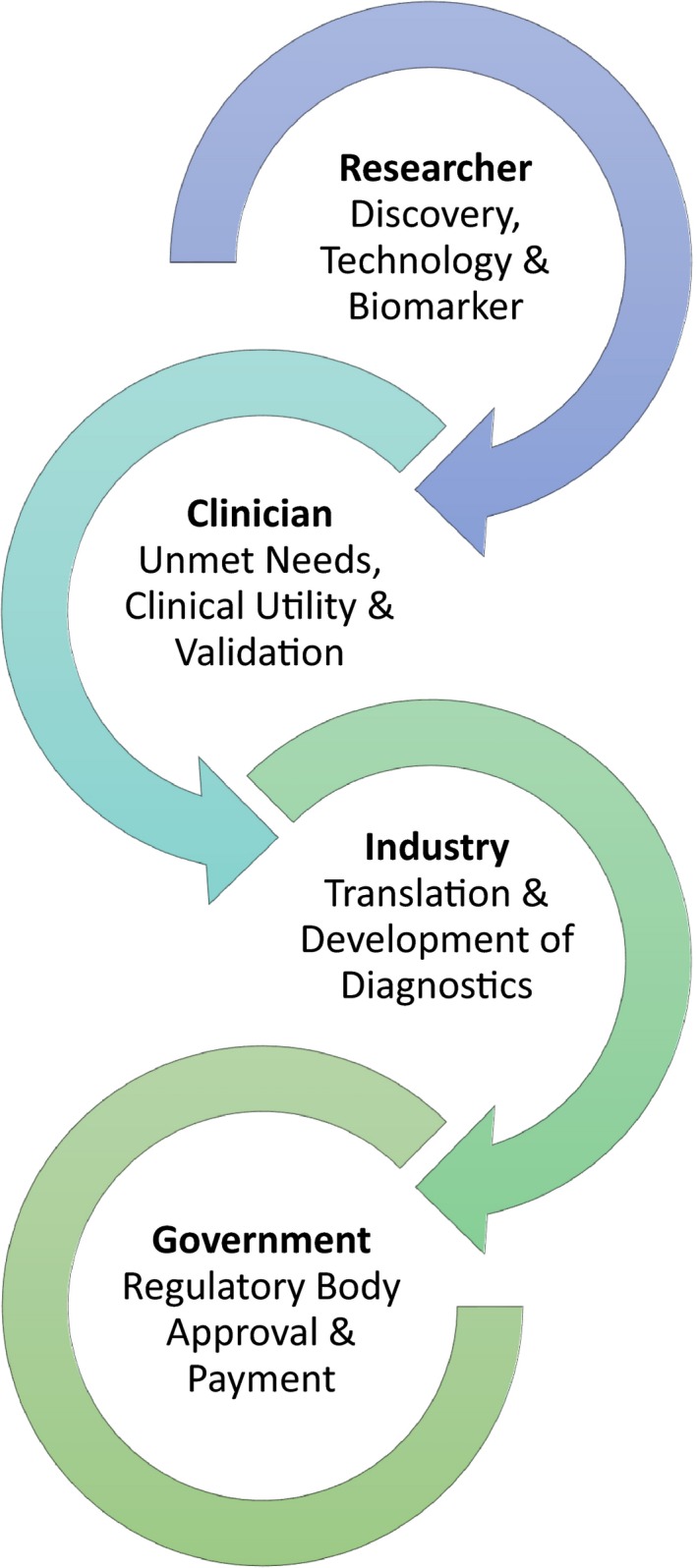
Flow diagram “Bench to Clinic”.

Highlighted in Figure 4, is the basic schematic of the different process leading to the diagnostic test developing from the laboratory to a clinical environment.

Reports suggest that the most efficient way to achieve acceptance of work into a clinical environment is to clearly highlight the current unmet needs and the requirement for the new clinical and intended uses. Presenting sufficient evidence in preliminary studies to support the investment for a large‐scale validation study, followed by the creation of methods with efficient analytical performance suitable for use in clinics would allow progression.

Finally, the design and implementation of clinical trials that allow for the demonstration of clinical usefulness help the research project to gain regulatory approval. All steps can be hard to achieve and the involvement of a multidisciplinary team is essential to ensure the accurate understanding of each process.

Focusing on the future diagnosis of brain tumors using spectroscopy and spectrometry methods via minimally invasive procedures, this review highlights the desperate need for advancement of current methodologies. The advantages of methods described are clear; quick analysis within a clinical setting, allowing patients to be treated earlier, and providing a better chance of survival. However, brain tumor diagnosis can only be revolutionized by addressing preanalytical factors, producing clinical trial plans, government approval, and understanding the translation into clinics prior to experimental proof‐of‐principle studies.

## Conflict of Interest

None declared.
